# Sex differences in self-report anxiety and sleep quality during COVID-19 stay-at-home orders

**DOI:** 10.1186/s13293-020-00333-4

**Published:** 2020-10-13

**Authors:** Jeremy A. Bigalke, Ian M. Greenlund, Jason R. Carter

**Affiliations:** 1 Department of Health and Human Development, Sleep Research Laboratory, Montana State University, Bozeman, MT 59717 USA; 2 Department of Psychology, Montana State University, Bozeman, MT USA; 3 Department of Kinesiology and Integrative Physiology, Michigan Technological University, Houghton, MI USA

**Keywords:** Anxiety, COVID-19, Pandemic, Sex, Sleep quality, Total sleep time

## Abstract

**Background:**

COVID-19 and home isolation has impacted quality of life, but the perceived impact on anxiety and sleep remains equivocal. The purpose of this study was to assess the impact of COVID-19 and stay-at-home orders on self-report anxiety and sleep quality, with a focus on sex differences. We hypothesized that the COVID-19 pandemic would be associated with increased anxiety and decreased sleep quality, with stronger associations in women.

**Methods:**

One hundred three participants (61 female, 38 ± 1 years) reported perceived changes in anxiety and sleep quality due to stay-at-home orders during the COVID-19 pandemic and were administered the Spielberger State-Trait Anxiety Inventory (STAI), Pittsburgh Sleep Quality Index (PSQI), and Insomnia Severity Index (ISI). Chi-square and *T* test analyses were utilized to assess sex differences in reported anxiety and sleep. Analysis of covariance was used to compare the associations between reported impact of COVID-19 and anxiety/sleep parameters.

**Results:**

Women (80.3%) reported higher prevalence of increased general anxiety due to COVID-19 when compared to men (50%; *p* = 0.001) and elevated STAI state anxiety compared to men (43 ± 1 vs. 38 ± 1 a.u., *p* = 0.007). Despite these differences in anxiety, the perceived impact of COVID-19 on PSQI was not different between sexes. However, when stratified by perceived changes in anxiety due to COVID-19, participants with higher anxiety responses to COVID-19 had higher ISI compared to those with no perceived changes in anxiety (9 ± 1 vs. 5 ± 1 a.u., *p* = 0.003). Additionally, participants who reported reduced sleep quality due to COVID-19 reported higher state anxiety (45 ± 1 a.u.) compared to those that perceived no change (36 ± 2 a.u., *p* = 0.002) or increased (36 ± 2 a.u., *p* < 0.001) sleep quality.

**Conclusion:**

COVID-19 and state-ordered home isolation was associated with higher anxiety and reduced sleep quality, with a stronger association in women with respect to anxiety.

## Background

Since its initial emergence in the Chinese city of Wuhan in late 2019, the coronavirus disease (COVID-19), caused by the severe acute respiratory syndrome coronavirus 2 (SARS-CoV-2), has drastically altered social structures around the world. The number of global COVID-19 cases has grown to nearly 6 million, with over 350,000 deaths according to a recent situation report by the World Health Organization [1]. In the USA alone, nearly 2 million confirmed COVID-19 cases and over 100,000 deaths have been associated with virus contraction and associated disease complications [2]. However, in addition to the immediate impact of COVID-19 on infected patients, recent attention has turned toward the potential impact of COVID-19 and state-ordered home lockdown on anxiety and sleep within infected individuals and the general population [3–6]. Relevant to the COVID-19 pandemic, anxiety and decreased sleep quality are associated with immune system dysfunction, which can increase susceptibility to infection [7–11]. Moreover, both anxiety and decreased sleep quality are associated with a number of cardiometabolic diseases, including diabetes [12, 13] and hypertension [14–16], which is important to note given that recent evidence suggests COVID-19 is associated with further detrimental vascular complications [17–20]. Lastly, anxiety and compromised sleep can exacerbate mental health risk, including higher prevalence of suicidal ideation [21, 22]. Accordingly, understanding the relationship between home isolation due to the COVID-19 pandemic, anxiety, and sleep is relevant to both short- and long-term health.

The bidirectional relationship between anxiety and sleep is well documented [23, 24] and comorbid with a variety of pathological conditions [12, 14–16, 25]. More recent studies have sought to define the overall impact of COVID-19 pandemic on general anxiety and sleep quality. An online analysis of the psychological impact of COVID-19 on Chinese citizens using the Impact of Event Scale-Revised showed that 53.8% of respondents reported moderate to severe psychological impact due to the virus, even when the spread of the virus was in its infancy and not yet classified as a pandemic [26]. More specifically, 30% reported suffering from some form of depression, while 37% reported heightened anxiety [26]. Similarly, a large-scale national survey performed in China with over 50,000 respondents showed that around 35% of those who responded reported some form of psychological distress [27], comparable to the findings of Huang and Zhao who reported a similar prevalence of generalized anxiety disorder [28]. The few studies assessing anxiety in the USA mimic these results, showing a significant association between stay-at-home orders and reported health anxiety levels [29], and elevated anxiety among young adults [30]. With regards to sleep, Huang and Zhao [28] observed an 18.2% prevalence of poor sleep quality as assessed by the Pittsburgh Sleep Quality Index (PSQI) [28], while a similar study performed by Zhao et al. [31] reported a nearly two-fold higher prevalence of PSQI-defined poor sleepers at 37% of the sampled population.

Despite recent evidence suggesting an association between COVID-19 lockdown and heightened anxiety and/or poor sleep in the Chinese population, to our knowledge, there have not yet been studies performed in the USA assessing these associations. Furthermore, studies to date have included conflicting reports on the impact of lockdown on anxiety and sleep in men versus women [26–30, 32]. This is important because anxiety and certain sleep disorders, such as insomnia, tend to be more prevalent in women [33]. While there have been some reports that serious COVID-19 infections and deaths tend to be more prevalent in male population, it is reasonable to hypothesize that the indirect impact of COVID-19 (i.e., home isolation) has a disparate impact on women, particularly with respect to anxiety and sleep.

Therefore, the purpose of this study was to assess the perceived impact of state-ordered home lockdown due to COVID-19 on anxiety and sleep quality in the USA, with a particular focus on differences between men and women. We hypothesized that the home lockdown would be associated with increased anxiety and ultimately decreased sleep quality and that these associations would be stronger in women compared to men.

## Methods

### Participants

All participants were sampled from areas that were under state-specific stay-at-home orders due to the COVID-19 pandemic between April 25 and May 18, 2020, approximately 6 weeks after COVID-19 was declared a pandemic by the World Health Organization, as well as a national emergency in the USA. Recruitment included word-of-mouth and online advertisement. Participants were excluded if the surveys were not completed during stay-at-home orders. In addition, no participants were allowed to be under home “quarantine” due to contraction of COVID-19 at the time of survey completion. All participants were adults between the ages of 18 and 70 years. Of the 127 total respondents, 22 were excluded due to incomplete survey responses and 2 were excluded due to state-specific stay-at-home orders being lifted during the time of the survey submission. The remaining 103 participants (42 male, 61 female, age: 38 ± 1, BMI: 27 ± 1 kg/m^2^) were primarily from Michigan (*n* = 61), Montana (*n* = 18), and Wisconsin (*n* = 15). Other respondents reported current residency in Indiana (*n* = 2), Minnesota (*n* = 2), New York (*n* = 1), Delaware (*n* = 1), Texas (*n* = 1), Massachusetts (*n* = 1), and Pennsylvania (*n* = 1). All participants provided voluntary electronic consent for participation in the study. Procedures and protocols used were approved by the Montana State University Institutional Review Board and in accordance with the Declaration of Helsinki.

### Study design

Participants filled out an initial battery of questionnaires through REDCap, an online confidential database. Participants were given a screening questionnaire to ensure study eligibility and to collect information on comorbid conditions, anthropometrics, demographics, occupation, and socioeconomic status. Validated “STOP-BANG” [34] questionnaires were also utilized to assess likelihood of having obstructive sleep apnea among the respondents for use as a covariate. A higher STOP-BANG score corresponds with a higher likelihood of having obstructive sleep apnea (OSA). Participants were then asked to answer a general questionnaire created by our research team to assess how participants perceived the impact of COVID-19 on sleep quality, mood, diet, physical activity, anxiety, alcohol consumption, and overall quality of life (i.e., “Please state how COVID-19 has impacted each of the following activities using this scale…”). A 5-point Likert scale was utilized with the following options: (1) greatly worsened/decreased, (2) somewhat worsened/decreased, (3) remained unchanged, (4) somewhat improved/increased, and (5) greatly improved/increased.

Next, participants were asked to fill out a number of validated surveys to subjectively measure their anxiety, depression, and sleep quality, including (1) Spielberger State and Trait Anxiety Inventory (STAI) [35], (2) Pittsburgh Sleep Quality Index (PSQI) [36], (3) Epworth Sleepiness Scale (ESS) [37], (4) Center for Epidemiological Studies Depression screen (CES-D) [38], and (5) Insomnia Severity Index (ISI) [39]. Lastly, participants were asked how the COVID-19 pandemic affected their occupation status and sleep schedule (i.e., typical bedtime vs. awakening).

### Anxiety and sleep questionnaires

#### Spielberger State-Trait Anxiety Inventory

The STAI [35] assesses self-reported anxiety (both state and trait anxiety) using a validated 40-item Likert scale questionnaire. State anxiety reflects transient (i.e., current moment) emotional anxiety due to situational stress. Trait anxiety assesses an individual’s predisposition to react with anxiety in any stressful event. Together, the STAI allows quantification of personal characteristic anxiety reactivity, as well as transient fluctuations dependent on the situation.

#### Pittsburgh Sleep Quality Index

The PSQI [36] is a validated subjective measure of sleep quality over the past month. The PSQI consists of 19 questions that offer a global sleep quality score. This global sleep quality score consists of 7 component scores assessing the following: sleep quality, sleep latency, sleep duration, habitual sleep efficiency, sleep disturbance, use of sleeping medications, and daytime dysfunction. A global PSQI > 5 arbitrary units represents poor sleep.

#### Epworth Sleepiness Scale

The ESS [37] evaluates daytime sleepiness using 8 questions. Respondents are asked to rate, on a validated 4-point scale, their usual chance of dozing during 8 different activities. The scale offers a general daily sleep propensity.

#### Center for Epidemiological Studies Depression Scale

The CES-D [38] is a validated 20-item scale where participants are asked to rate depressive symptoms on a scale of 0–3, with scores above 16 suggesting clinically significant depression.

#### Insomnia Severity Index

The ISI [39] is a 7-item survey assessing any potential functional impact of insomnia. Participants use a validated 0–4 point rating, and questions pertain to the last 2 weeks. The total ISI is the sum of all question points, and any score above 7 corresponds to some level of insomnia symptoms.

### Statistical analysis

All data was analyzed using commercially available statistical software (SPSS 25.0; SPSS, Chicago, IL). Chi-square analysis was performed to assess any associations between gender and responses to our subjective general questionnaire. In order to meet the assumption for chi-square analysis that the cell expected frequency count should be 5 or more in at least 80% of cells [40], we combined those individuals who reported “decreased” or “unchanged” anxiety, as well as those who reported “increased” or “unchanged” sleep quality for analysis. Comparisons of baseline characteristics between sexes (male vs. female) were performed using an independent samples *T* test. Analysis of covariance (ANCOVA) testing was performed to compare differences in characteristics between groups stratified based upon perceived change in anxiety, sleep quality, or total sleep time (TST) (i.e., decreased, unchanged, or increased) while controlling for age, BMI, occupation status (unemployed, unchanged status, working from home, temporarily/permanently laid-off), and STOP-BANG questionnaire scores as covariates. All data are expressed as mean ± standard error unless otherwise noted. In the case of ANCOVA analysis, the mean adjusted for covariance is presented in the results. If a significant interaction was observed, Bonferroni adjusted pairwise analysis was performed for post hoc analysis between adjusted means. In those tests that a significant interaction or difference was observed, Cohen’s D and partial eta squared tests of effect size in *T* tests and ANCOVA analyses are reported. A significance level of *α* = 0.05 was set for all statistical tests.

## Results

### Summary data from entire sample

Table 1 shows that the majority of the 103 participants reported decreased/worsened sleep quality (56.3%), daily schedule (68.9%), and overall quality of life (58.3%). Respondents reported increased anxiety (68.0%) and increased time spent in front of an electronic screen (77.7%). Using a cut-off of PSQI > 5 arbitrary units (a.u.), 66% of the population qualified for classification as “poor sleepers,” while 47.6% reported some signs of at least mild insomnia symptoms (ISI > 7).

**Table 1:** Response rates to lifestyle impact of COVID-19

Parameter	
Decrease (%)	
Sleep quality	56.3
Physical activity	46.6
Quality of life	58.3
Increase (%)	
Anxiety	68.0
Screen time	77.7
Alcohol consumption	34
Desire to consume alcohol	39.8
Worsened (%)	
Mood	49.5
Diet	35
Daily schedule	68.9

Percentage of participants (*n* = 103) who reported detriment in the listed lifestyle parameters

Furthermore, 66 individuals (64%) reported having a full-time position prior to the COVID-19 pandemic and stay-at-home orders. Approximately 88% of individuals that were employed prior to COVID-19 reported that they were now working from home (*n* = 42) or that their job had remained unchanged (*n* = 16), while 12% reported temporary or permanent layoff (*n* = 8) due to the pandemic.

### COVID-19, anxiety, and sleep: sex differences

**Fig. 1:**
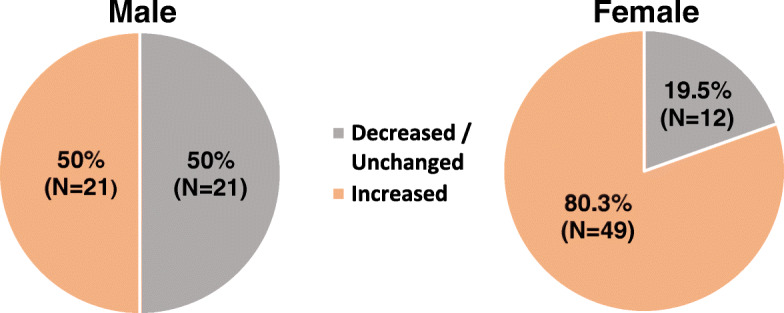
Chi-square analysis of sex and perceived changes in anxiety due to COVID-19. The proportion of men and women who reported increased versus unchanged/decreased anxiety due to COVID-19 and state-ordered home quarantine

Table 2 demonstrates baseline characteristics of the sample respondents, as well as differences between males and females as regards to anthropometrics, occupation status, and survey responses. Females reported a heightened state anxiety compared to men (43 ± 1 vs. 38 ± 1 a.u., *p* = 0.007, *d* = 0.55). However, all other anxiety and sleep parameters were not significantly different between sexes. Chi-square (*χ*^2^) analysis showed a significant relationship between sex and respondents’ perceived changes in anxiety (*χ*^2^ (1, *N* = 103) = 10.507, *p* = 0.001), but not sleep (*χ*^2^ (1, *N* = 103) = 0.069, *p* = 0.793), during COVID-19 lockdown. Figure 1 depicts the disproportionate number of women with higher perceived anxiety during COVID-19 compared to men.

**Table 2:** Baseline characteristics

Variable	Male	Female	*P* value	All
*N* (%)	42 (41)	61 (59)	---	103 (100)
Age (Range)	37 ± 2 (18-68)	39 ± 2 (19-68)	0.393	38 ± 1 (18-68)
BMI	28 ± 1	26 ± 1	0.058	27 ± 1
STOP-BANG	3 ± 0	1 ± 0	<0.001	2 ± 0
Sleep disorder	1	1	---	2
Employment status, *N* (%)				
Unemployed	14 (37.8)	23 (62.2)	---	37 (35.9)
Unchanged	8 (50)	8 (50)	---	16 (15.5)
Working from home	17 (40.5)	25 (59.5)	---	42 (40.8)
Laid-off	3 (37.5)	5 (62.5)	---	8 (7.8)
STAI				
State	38 ± 1	43 ± 1	0.007	41 ± 1
Trait	37 ± 1	39 ± 1	0.284	38 ± 1
ESS	5 ± 1	5 ± 0	0.331	5 ± 0
CES-D	11 ± 1	15 ± 1	0.061	13 ± 1
ISI	8 ± 1	7 ± 1	0.702	7 ± 0
PSQI	7 ± 0	7 ± 0	0.854	7 ± 0

Subject baseline characteristics in men and women. Values are mean ± SEM unless otherwise specified. Percentage values in male and female employment status are representative of the proportion of each sex that make up each employment category

*BMI* body mass index, *STAI* State-Trait Anxiety Inventory, *ESS* Epworth Sleepiness Scale, *CES-D* Center for Epidemiological Studies Depression Scale, *ISI* Insomnia Severity Index, *PSQI* Pittsburgh Sleep Quality Index

### Stratification by perceived change in anxiety

**Fig. 2:**
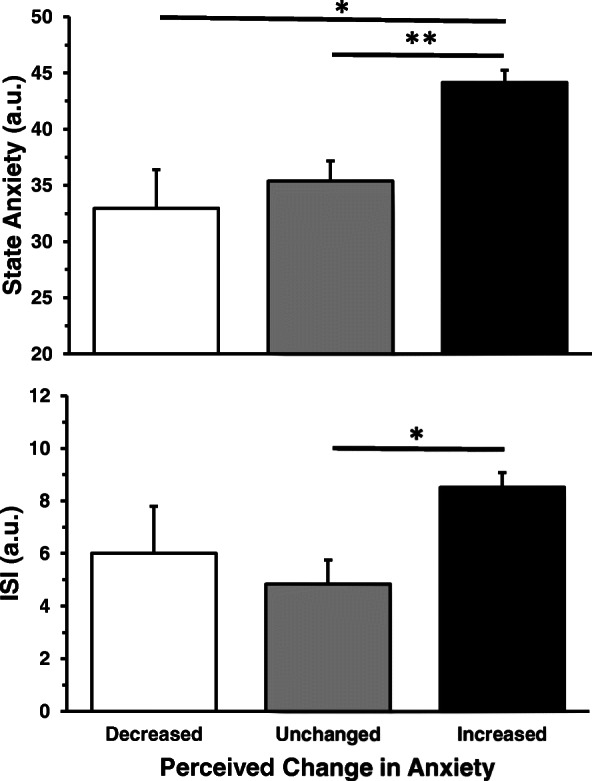
Anxiety and sleep parameters stratified by perceived changes in anxiety due to COVID-19. State anxiety and Insomnia Severity Index (ISI) mean covariate adjusted scores in those who reported decreased, unchanged, or increased anxiety due to COVID-19. Decreased anxiety, *N* = 7; unchanged anxiety, *N* = 26; increased anxiety, *N* = 70. *P < 0.01; **P < 0.001. a.u., arbitrary units

Participants were stratified into groups based on whether they reported decreased (*n* = 7), unchanged (*n* = 26), or increased anxiety (*n* = 70) due to the COVID-19 pandemic. There was a significant interaction between anxiety stratification and the dependent variables of state anxiety (F(2,100) = 11.577, *p* < 0.001, η_p_^2^ = 0.194) and ISI (F(2,100) = 6.046, *p* = 0.003, η_p_^2^ = 0.112), but not PSQI (F(2,100) = 1.458, *p* = 0.238). As depicted in Fig. 2, those who reported a perceived increase in anxiety due to COVID-19 home lockdown had higher state anxiety (44 ± 1 a.u.) when compared to those that perceived no change (35 ± 2 a.u., *p* < 0.001) or decreased (33 ± 3 a.u., *p* = 0.008) anxiety during COVID-19 lockdown. Figure 2 also highlights that those who reported increased anxiety had higher ISI scores (9 ± 1 a.u.) than those who reported unchanged anxiety (5 ± 1 a.u., *p* = 0.003), while there was no difference compared to those who reported decreased anxiety (6 ± 2 a.u., *p* = 0.555).

### Stratification by perceived change in sleep quality

**Fig. 3:**
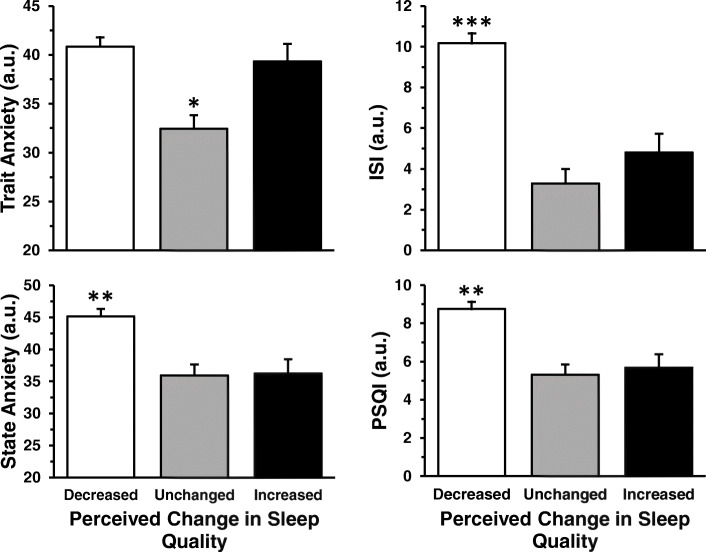
Anxiety and sleep parameters stratified by perceived changes in sleep quality due to COVID-19. Trait anxiety, state anxiety, Insomnia Severity Index (ISI), and Pittsburgh Sleep Quality Index (PSQI) in those who reported decreased, unchanged, or increased sleep quality (SQ) due to COVID-19. Decreased SQ, *N* = 58; unchanged SQ, *N* = 28; increased SQ, *N* = 17. *P < 0.05 vs. groups; **P < 0.001 vs. all groups. a.u., arbitrary units

Figure 3 shows STAI, CES-D, ISI, and PSQI data stratified by participants who perceived that sleep quality was decreased (*n* = 58), unchanged (*n* = 28), or increased (*n* = 17) due to COVID-19 home quarantine. There was a significant interaction between group stratification and the dependent variables state anxiety (F(2,100) = 12.747, *p* = < 0.001, η_p_^2^ = 0.210), trait anxiety (F(2,100) = 12.712, *p* < 0.001, η^2^ = 0.209), ISI (F(2,100) = 36.829, *p* < 0.001, η_p_^2^ = 0.434), and PSQI (F(2,100) = 16.665, *p* < 0.001, η_p_^2^ = 0.258). Post hoc analyses revealed state anxiety was significantly higher in those who reported decreased sleep quality (45 ± 1 a.u.) when compared to those who reported increased (36 ± 2 a.u., *p* = 0.002) and unchanged (36 ± 2 a.u., *p* < 0.001) sleep quality during home isolation. In contrast, trait anxiety was lower in those who reported unchanged sleep quality (32 ± 1 a.u.) when compared to those who reported perceived either decreases (41 ± 1 a.u., *p* < 0.001) or increases (39 ± 2 a.u., *p* = 0.009) in sleep quality due to COVID-19 lockdown. Those who reported perceived decreases of sleep quality due to COVID-19 lockdown had a significantly increased ISI (10 ± 0 a.u.) and PSQI (9 ± 0 a.u.) when compared to those who reported either unchanged (ISI: 3 ± 1 a.u., *p* < 0.001; PSQI: 5 ± 1 a.u., *p* < 0.001) or increased (ISI: 5 ± 1 a.u., *p* < 0.001; PSQI: 6 ± 1 a.u., *p* = 0.001) sleep quality due to COVID-19 home lockdown.

### Stratification by self-report total sleep time

**Fig. 4:**
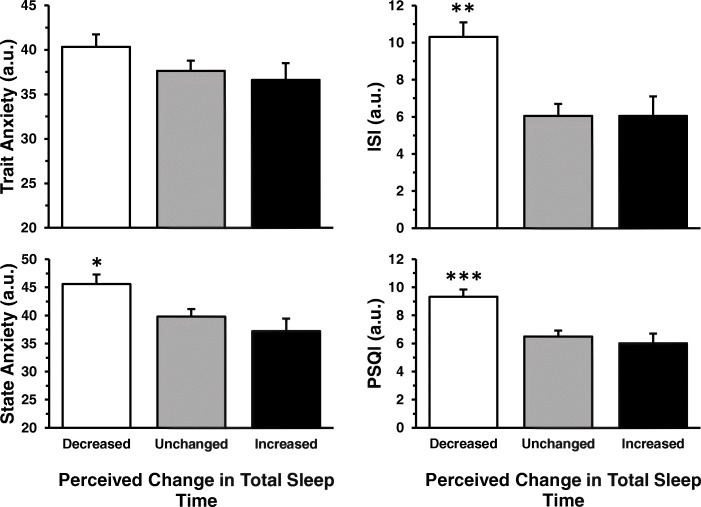
Anxiety and sleep parameters stratified by perceived changes in total sleep time (TST) due to COVID-19. Trait anxiety, state anxiety, Insomnia Severity Index (ISI), and Pittsburgh Sleep Quality Index (PSQI) in those who were determined to have decreased, unchanged, or increased TST due to COVID-19. Decreased TST, *N* = 33; unchanged TST, *N* = 51; increased TST, *N* = 19. *P < 0.05 vs. all groups. **P < 0.01 vs. all groups. ***P ≤ 0.001 vs. all groups. a.u., arbitrary units

Figure 4 depicts STAI, CES-D, ISI, and PSQI data stratified by participants who perceived that self-report total sleep time was decreased (*n* = 33), unchanged (*n* = 51), or increased (*n* = 19) during the COVID-19 pandemic. There was a significant interaction between TST groupings and the dependent variables state anxiety (F(2,100) = 5.613, *p* = 0.005, η_p_^2^ = 0.105), ISI (F(2,100) = 9.789, *p* < 0.001, η_p_^2^ = 0.169), and PSQI (F(2,100) = 10.877, *p* < 0.001, η_p_^2^ = 0.185), but not trait anxiety (F(2,100) = 1.608, *p* = 0.206). Post hoc analyses revealed that state anxiety was elevated in those who perceived a decreased TST (46 ± 2 a.u.) due to COVID-19 lockdown when compared to those who reported unchanged (40 ± 1 a.u., *p* = 0.028) or increased (37 ± 2 a.u., *p* = 0.010) TST. Similarly, those who reported a decrease in TST also reported a higher ISI (10 ± 1 a.u.) and PSQI (9 ± 1 a.u.) when compared to those who reported an unchanged (ISI: 6 ± 1 a.u., *p* < 0.001; PSQI: 6 ± 0 a.u., *p* < 0.001) or increased (ISI: 6 ± 1 a.u., *p* = 0.005; PSQI: 6 ± 1 a.u., *p* = 0.001) TST during the COVID-19 stay-at-home orders.

## Discussion

The COVID-19 pandemic has had a major impact on human health globally. Aside from its immediate effects on those infected by the virus, the COVID-19 stay-at-home orders may have adverse effects on both anxiety and sleep parameters, which can exacerbate comorbid illnesses. The present study sought to assess the impact of state-ordered home lockdown on perceived anxiety and sleep, with a focus on potential differences between men and women. We report four novel findings. First, a disproportionately greater number of women reported that they perceived a greater increase in anxiety directly due to home isolation, and women also demonstrated higher levels of situational (i.e., state) anxiety compared to men. Second, when data were stratified by participants’ perceived changes in anxiety due to COVID-19 lockdown, there was a significant relationship between higher perceived anxiety and insomnia symptoms assessed by ISI, but not with sleep quality assessed by PSQI. Third, those who reported a decrease in perceived sleep quality due to COVID-19 reported significantly higher state anxiety, insomnia symptoms, and poorer sleep quality when compared to those whose sleep quality was reportedly unchanged or increased due to stay-at-home orders. Finally, those whose TST was reduced during COVID-19 lockdown based on the comparison of their current and past self-report TST presented higher anxiety and insomnia symptoms, as well as reduced sleep quality, when compared to those with unchanged or increased TST during the COVID-19 home lockdown. These findings are the first to highlight significant associations between stay-at-home orders, anxiety, and poor sleep in the USA and that women have stronger associations with regard to anxiety.

Anxiety and sleep disturbances are often described as comorbid conditions [23, 24]. It is well established that chronic short and/or poor sleep quality increases risk of hypertension [14–16], autonomic nervous system dysregulation [41, 42], atherosclerosis [25], and diabetes [12]. Similarly, severe anxiety and reduced mental health are known contributors to poor cardiometabolic outcomes [43]. Since the early reports of the COVID-19 pandemic in China [44], and following the virus spread in the USA and the globe [45], a disproportionate percentage of individuals with preexisting conditions die from complications associated with COVID-19. This is believed to be, in part, related to how the SARS-CoV-2 virus binds to the Angiotensin-converting-enzyme-2 (ACE2) receptor, which is present within the vasculature and lungs [46]. The expression of this receptor is upregulated in individuals with diabetes and hypertension, a common target for pharmacological treatment [47].

Previous disease outbreaks have offered insight in to the impact that diseases have on quality of mental health [48–50]. A recent meta-analysis of 25 different studies performed on infected individuals during outbreaks of severe acute respiratory syndrome (SARS) and Middle East Respiratory Syndrome (MERS), two viruses within the coronavirus family, showed that individuals who were infected suffered serious acute, and even some lingering effects on anxiety and poor sleep [48]. Specifically, 35.7 and 41.9% of those hospitalized exhibited acute symptoms of anxiety and insomnia [48], which is similar to our results specific to COVID-19. However, this prior meta-analysis was performed on individuals who were suspected or confirmed to have been infected by either SARS or MERS.

Recent reports from China point to both elevated anxiety and decreased sleep quality among medical workers [32, 51] and the general public [26–28] in response to COVID-19. Even prior to classification as a pandemic, Wang et al. found that over 50% of participants reported a moderate to severe psychological impact of COVID-19 home quarantine in a study of 1210 Chinese citizens [26]. A recent review by Brooks and colleagues [3] outlines some of the primary causative factors for excessive stress during quarantine, including (1) duration of quarantine, (2) fear of infection, (3) frustration or boredom, (4) inadequate supplies, and (5) inadequate information. These factors are relevant to the present study because all of our participants were sampled from states under state orders for home isolation, and thus were likely experiencing some, if not all, of the aforementioned stressors. This is reflected in the proportion of our study population that reported a significant impact of COVID-19 and stay-at-home orders on their physical and emotional well-being.

In the USA, the COVID-19 pandemic has presented unprecedented social restrictions to nearly all generations, with severely restricted travel and in-person social interaction to help reduce the disease progression. A recent study performed during stay-at-home orders in the USA found that increased anxiety was independently associated with implementation of stay-at-home restrictions [29]. Interestingly, two studies by Xiao et al. [52, 53] have implied that social isolation and social capital directly impact anxiety, but not sleep quality, in healthcare professionals [52] and individuals under a 14-day self-quarantine due to potential contact with COVID-19 [53]. However, the authors suggest that anxiety acts as a mediator between social isolation and sleep impairment in these populations. Likewise, in a study of 1630 Chinese citizens, Zhao et al. [31] reported that subjective anxiety levels accounted for 66% of the total effect of stress on sleep quality. Collectively, these studies indicate a relationship between social quarantine and both increased anxiety and decreased sleep quality.

Recent studies from the USA and China included conflicting reports regarding the impact of sex on COVID-19 and anxiety. Specifically, some reported increased psychological susceptibility in women during the COVID-19 pandemic [27, 29, 32], while others reported increased anxiety and stress in Chinese men [26], or no differences between sexes [28, 30]. More research is necessary to determine whether women may be more susceptible to the negative psychological impacts of COVID-19 home lockdown. In the present study, we found that women reported a greater increase in anxiety due to state-ordered home isolation when compared to men and that women also demonstrated higher levels of situational (i.e., state) anxiety. This is consistent with a large body of research reporting increased prevalence and risk of anxiety disorders among women [54, 55]. It is quite possible that females reported higher anxiety due to a predisposition toward higher anxiety levels, as females show a higher prevalence of anxiety disorders compared to males. However, we did not observe any sex differences in trait anxiety, which is an accurate measure of trait predisposition toward higher anxiety, in the present population. Furthermore, we assessed changes in occupation status and found that there were no significant sex-related changes in occupation status following COVID-19 state-ordered lockdown. While we did not find differences in occupation status, it is possible the familial responsibilities associated with females who were either unemployed or working from home were more strenuous than those of males. Closure of schools throughout the country resulted in a large majority of parental figures having to not only work from home but also care for their children’s emotional, physical, and intellectual well-being during a time when they would normally be in a school setting. A recent study performed between mid-to-late April found that in a population of over 600 mothers during the beginning of the COVID-19 pandemic, rates of anxiety and depression were elevated in the context of COVID-19 compared to the normal non-pandemic population averages [56]. While we did not assess parental responsibility directly in our survey, we allowed participants to freely respond with any extraneous variables that may be impacting their anxiety and sleep quality. While not all participants chose to respond to these open-ended questions, 20 (~ 19%) respondents reported concern over mounting parental responsibilities, or worries concerning family well-being. Of these 20 respondents, 14 (70%) were female, suggesting the potential for heightened concern in females compared to males, which may exacerbate anxiety levels. Furthermore, we observed elevated, although non-significant, depression levels (CES-D) in females compared to males (Table 2). These findings are similar to those presented by Cameron and colleagues [56], who showed a positive, bidirectional correlation between depression and anxiety in mothers during COVID-19. Although the direct cause of the observed disparity in anxiety symptoms is not known, the relationship between sex and anxiety is relevant to how we manage future COVID-19 or other disease outbreaks and state-ordered lockdowns.

Contrary to our hypothesis, we did not observe any differences in sleep quality as measured by the PSQI in those who reported increased perceived anxiety due to COVID-19 lockdown. Similarly, when those who reported decreased or unchanged were paired together and compared to those with high anxiety, we still found no significant difference in PSQI (data not shown). We postulate that the COVID-19 pandemic may have offered an increased sleep opportunity and less responsibilities to some individuals studied due to the home quarantine orders. Although we did not directly ask questions concerned with sleep opportunity, around 30–40% of the general population in the USA has previously reported short sleep duration and opportunity in three national surveys, including the Behavioral Risk Factor Surveillance System (BRFSS) [57], National Health and Nutrition Examination Survey (NHANES) [58], and the National Health Interview Survey – Sample Adult Files (NHIS-SAF) [59].

In contrast to the PSQI, we did find that the ISI scores were higher in those individuals who reported increased anxiety due to home lockdown. It is worth noting again that the proportion of those who reported increased anxiety were women. Women have consistently been shown to report increased or more severe insomnia symptoms when compared to men [33]. However, we did not find any differences in ISI when comparing men and women. Although the increased anxiety group was primarily comprised of females, we do not believe this was driving the finding due to this reason. Previous literature has shown a bidirectional relationship between insomnia and anxiety [24]. Furthermore, higher reactivity and vulnerability to stressors has been shown to predispose some individuals to the development of insomnia [60, 61]. Our results may point to an underlying trait disposition that predisposes those individuals who reported heightened anxiety during state-ordered lockdown to increased insomnia symptoms. The elucidation of these risk factors that predispose an individual to further mental health and sleep complications in response to lockdown in the context of COVID-19 may offer a means of more efficient mental health treatment during the pandemic and any future outbreaks.

### Limitations

We acknowledge a number of limitations with our study design. First, this study serves as a snapshot of the perceived impacts that the COVID-19 pandemic has on both sleep and anxiety. It will be imperative for other research groups to include longitudinal aspects in their study design. As the virus spreads and home isolation orders change, more causative reasoning can be applied to why anxiety and sleep disturbances may be occurring if sampled multiple times. Second, the majority of this study was of Caucasian/European decent. Numerous reports regarding COVID-19 spread and disease severity in the USA demonstrate the African-American and Hispanic communities have been disproportionately affected [62]. More research is desperately needed in this area, as cardiovascular disease prevalence is also highest in African-American and Hispanic individuals [63]. Third, a degree of convenience sampling was used to solicit this study, with majority representation from Michigan, Wisconsin, and Montana. Many other states (i.e., California, New Jersey) with more dense population centers saw increased hospitalizations and deaths associated with COVID-19; thus, it is possible that the impact of COVID-19 home quarantine on perceived anxiety and sleep quality may be different based on the geographical region assessed. In addition, cognitive capacity and ability were not evaluated in this study. This is important given the wide age range of the study. However, age was included as a covariate to reduce the variance. Finally, a recent study by Altena et al. [64] highlighted the new demands of working from home and specifically mentioned the demands that many women face also spending a significant amount of time helping their children with school-related homework. It is plausible that these demands may have disproportionally impacted women, but we did not ask for specific details on family and/or caregiving status. Future work should account for these additional factors.

### Perspectives and significance

The present study assessed differential impacts of COVID-19 and home isolation on sleep quality and anxiety in men and women by utilizing validated surveys and self-report assessment. To date, no research studies have assessed the impact of COVID-19 and home isolation on anxiety and sleep disturbance, with specific comparisons of sex differences in the USA. We found that the majority of individuals reported worsened anxiety and sleep quality, but females reported a higher prevalence of increased anxiety due to COVID-19. Furthermore, heightened anxiety was associated with increased insomnia symptoms. These findings offer insight into the differential impact of a global pandemic and social isolation on mental health and sleep parameters in men and women, with implications for worsened mental health in women. These findings indicate the need for adequate mental health services, especially in times of social isolation. In addition, sleep detriment may be largely associated with mental health detriment during global pandemics and restricted living. Future research should utilize objective sleep measurements, such as actigraphy. Our findings provide novel insight into the interplay between anxiety and sleep quality, with specific consideration toward sex differences.

## Conclusions

Our results show that females disproportionately reported higher levels of perceived anxiety due to COVID-19 lockdown when compared to men. Furthermore, those who reported increased anxiety showed increased insomnia symptoms. Lastly, those who reported decreased sleep quality and quantity due to COVID-19 lockdown showed increased anxiety, insomnia symptoms, and decreased sleep quality. Our findings point to an overall increase in anxiety, and decrease in sleep quality and quantity, due to the COVID-19 pandemic and home lockdown, with specific sex differences in perceived anxiety.

## Data Availability

The datasets used and/or analyzed during the current study are available from the corresponding author on reasonable request.
